# DDIT3 is associated with breast cancer prognosis and immune microenvironment: an integrative bioinformatic and immunohistochemical analysis

**DOI:** 10.7150/jca.96491

**Published:** 2024-05-28

**Authors:** Xin Yu, Wenge Li, Shengrong Sun, Juanjuan Li

**Affiliations:** 1 Department of Breast and Thyroid Surgery, Renmin Hospital of Wuhan University, Wuhan, Hubei, P. R. China.; 2 Department of Oncology, Shanghai GoBroad Cancer Hospital, Shanghai, P. R. China.; 3 Department of general surgery, Taikang Tongji (Wuhan) Hospital, Wuhan, Hubei, P. R. China.

**Keywords:** breast cancer, bioinformatics, DDIT3, biomarker, therapeutic target

## Abstract

DNA damage-inducible transcript 3 (DDIT3) is a transcription factor central to apoptosis, differentiation, and stress response. DDIT3 has been extensively studied in cancer biology. However, its precise implications in breast cancer progression and its interaction with the immune microenvironment are unclear. In this study, we utilized a novel multi-omics integration strategy, combining bulk RNA sequencing, single-cell sequencing, spatial transcriptomics and immunohistochemistry, to explore the role of DDIT3 in breast cancer and establish the correlation between DDIT3 and poor prognosis in breast cancer patients. We identified a robust prognostic signature, including six genes (unc-93 homolog B1, TLR signaling regulator, anti-Mullerian hormone, DCTP pyrophosphatase 1, mitochondrial ribosomal protein L36, nuclear factor erythroid 2, and Rho GTPase activating protein 39), associated with DDIT3. This signature stratified the high-risk patient groups, characterized by increased infiltration of the regulatory T cells and M2-like macrophages and fibroblast growth factor (FGF)/FGF receptor signaling activation. Notably, the high-risk patient group demonstrated enhanced sensitivity to immunotherapy, presenting novel therapeutic opportunities. Integrating multi-omics data helped determine the spatial expression pattern of DDIT3 in the tumor microenvironment and its correlation with immune cell infiltration. This multi-dimensional analysis provided a comprehensive understanding of the intricate interplay between DDIT3 and the immune microenvironment in breast cancer. Overall, our study not only facilitates understanding the role of DDIT3 in breast cancer but also offers innovative insights for developing prognostic models and therapeutic strategies. Identifying the DDIT3-related prognostic signature and its association with the immune microenvironment provided a promising avenue for personalized breast cancer treatment.

## Introduction

Breast cancer is the most common malignancy affecting women worldwide; it exerts a considerable impact on public health and the quality of life 1. Despite substantial progress in breast cancer research, researchers should identify and characterize novel biomarkers that can serve as therapeutic targets or diagnostic tools 2. Understanding the underlying molecular mechanisms and key players in breast cancer development and progression is crucial to improving patient outcomes 1,2.

DNA damage-inducible transcript 3 (DDIT3), also termed CCAAT/enhancer-binding protein homologous protein, is a promising candidate 3. DDIT3 encodes a transcription factor central to various cellular processes, including apoptosis, differentiation, and stress response 4. DDIT3 dysregulation has been implicated in several diseases, including cancer 3. It is involved in multiple signaling pathways and cellular processes relevant to tumorigenesis. Additionally, DDIT3 regulates cell cycle progression 5, DNA repair 6, and apoptosis 6, suggesting its dynamic control over cellular homeostasis. Moreover, researchers have reported the role of DDIT3 in cancer biology, particularly in breast cancer 3,7.

Previously, researchers have demonstrated the dual role of DDIT3 in malignant tumors. DDIT3 is a central mediator of apoptosis associated with endoplasmic reticulum (ER) stress; various antitumor agents trigger cell death by activating DDIT3 8-11. Nonetheless, tumor cells can selectively activate DDIT3 through endogenous cellular and tumor microenvironment stressors, thereby facilitating tumor cell invasion, metastasis 12,13, angiogenesis 14, and immune evasion 15. However, the impact of DDIT3 on breast cancer prognosis and its underlying mechanisms are unclear. Hence, researchers should reassess the influence of DDIT3 on breast cancer.

In this study, we aimed to utilize bioinformatics to analyze the role of DDIT3 in breast cancer. We leveraged publicly available datasets and bioinformatics tools to offer novel insights into the molecular mechanisms underlying DDIT3-mediated effects in breast cancer progression. The outcomes can elucidate new therapeutic objectives and promote tailored therapeutic approaches for individuals with breast cancer.

## Materials and methods

### Patients and specimens

TCGA-BRCA cohort: RNA sequencing (RNA-seq) data and clinical records were obtained from 1,091 patients diagnosed with breast cancer, sourced from The Cancer Genome Atlas (TCGA) through the Xena data portal of the University of California Santa Cruz. Somatic mutation data were acquired using the R package TCGAbiolinks 16 and subsequently formatted as the Mutation Annotation Format for further analysis utilizing the R package maftools 17.

Molecular Taxonomy of Breast Cancer International Consortium cohort: RNA-seq data and clinical records obtained from 1,904 patients with breast cancer were downloaded from the Molecular Taxonomy of Breast Cancer International Consortium (METABRIC) databases (available at http://molonc.bccrc.ca/aparicio-lab/research/metabric/).

Immunotherapy-related cohort: RNA-seq data and clinical information of 348 patients with bladder cancer who received immunotherapy were obtained using the R package IMvigor210CoreBiologies 18.

We included all patients from the TCGA-BRCA cohort, METABRIC cohort, and IMvigor210 cohort that possess transcriptomic sequencing data and clinical information. This step can be attributed to the lack of explicit exclusion criteria for patients with comorbidities and associated information on other diseases within the original datasets. Additionally, cases with mRNA expression of DDIT3 above the median in their cohort were deemed to exhibit high DDIT3 expressions.

Immunohistochemistry (IHC) cohort: From February 2015 to August 2017, formalin-fixed paraffin-embedded specimens were collected from 129 women diagnosed with breast cancer at the Renmin Hospital of Wuhan University. Only patients with a follow-up period ≥5 years were included. Written informed consent was obtained from all the patients. The study was approved by the Institutional Ethics Committee of the Renmin Hospital of Wuhan University. The endpoint was defined as either recurrence or distant metastasis. The inclusion criteria were as follows: (1) confirmed by pathology; (2) no prior treatment, including chemotherapy, radiotherapy, or immunotherapy, before resection. The exclusion criteria were as follows: (1) with other concurrent diseases, (2) substantial organ dysfunction, and (3) with primary tumors in other organs.

Tables 1 to 3 summarize the patient characteristics in TCGA-BRCA, METABRIC, and IHC cohorts. Data from the public IMVIGOR210 cohort lack clinical characteristics, such as age and tumor clinical staging; therefore, they have not been listed.

### Immunohistochemistry

As outlined previously 19, IHC staining was conducted utilizing a framework following these steps: deparaffinization, antigen retrieval, endogenous peroxidase quenching, serum blocking (3% bovine serum albumin at room temperature for 30 min), overnight incubation with primary antibodies [DDIT3 (Proteintech, 15204-1-AP, 1/100)] at 4°C, and 50-min incubation with secondary antibody at room temperature. 3, 3'-diaminobenzidine was used for the staining, and the process was carefully monitored under a microscope. Subsequently, nuclear counterstaining was conducted by immersing the samples in a hematoxylin solution for approximately 3 min. The outcomes were evaluated based on the relative abundance and intensity of the positively stained neoplastic cells. In case of no prior clinical or follow-up data, two histopathologists examined DDIT3 protein expression independently using a multi-head microscope. The staining intensity was classified into four scores as follows: 0 indicating no staining, 1 indicating a faint, light brown shade, 2 indicating a moderate, brown shade, and 3 indicating a intense, dark brown shade. The protein expression score was calculated as follows: Total score = the proportion of positive cells × the depth of staining score. Cases with a protein expression score above the median were deemed to exhibit high DDIT3 expressions.

### Acquiring DDIT3-related genes

To investigate the association between DDIT3 and breast cancer, we conducted an in-depth analysis of gene expression data utilizing the Weighted Gene Co-expression Network Analysis (WGCNA) package 20. To discern the genes exhibiting the highest correlation with DDIT3, a comprehensive examination was conducted. Initially, the samples were clustered to assess the overall implication of each sample within the dataset; the outliers were subsequently excluded. The optimal soft thresholding power β was determined by identifying the lowest power value that substantially increased the scale-free topology fit index. Additionally, we conducted meticulous correlation analyses between various modules and phenotypic traits to identify the modules that were strongly associated with DDIT3.

### Biological functional analysis

We utilized the R package ClusterProfiler 21 to conduct an enrichment analysis of Gene Ontology (GO) and Kyoto Encyclopedia of Genes and Genomes (KEGG). We utilized the Gene Set Enrichment Analysis (GSEA) method to investigate gene set enrichment derived from the KEGG, GO, and Hallmark gene set (obtained from the Molecular Signatures Database (https://www.gsea-msigdb.org)) regarding gene expression. All analyses were performed using the R package ClusterProfiler.

### Constructing and validating a DDIT3-related prognostic signature

The DDIT3-related prognostic signature was constructed based on data obtained from the TCGA-BRCA study. Initially, univariate Cox regression analysis was conducted to identify the genes significantly associated with the overall survival (OS) within the modules related to DDIT. Least Absolute Shrinkage and Selection Operator (LASSO) regression analysis was conducted using the “glmnet” package in R 22. A 10-fold cross-validation procedure was implemented to select the most pertinent candidate genes for constructing the prognostic signature. This process facilitated determining the most important genes using LASSO regression. Furthermore, the association between the survival-associated genes and OS was evaluated using multivariate Cox regression analysis, which facilitated determining the multiple regression model and associated regression coefficients. The DDIT3-related prognostic signature was established as follows: Risk score = Σn1 coefi*xi. By considering the expression of the identified genes, a specific formula was applied to calculate the individual patient scores indicative of their risk level. These risk scores were utilized to classify the patients into high- or low-risk groups by setting the median risk score as the threshold. Patients with risk scores above and below the threshold were categorized into high- and low-risk groups, respectively. To identify the independent prognostic markers, a multivariate Cox analysis was conducted, considering both the risk score and clinical variables. Subsequently, a nomogram was constructed using the regplot software. It incorporated the age, stage, and risk score groups as the parameters. Calibration plots were generated to visually assess the consistency between the predicted and actual 1-, 3-, and 5-year OS rates.

### Computing enrichment scores of the gene signatures

By leveraging transcriptomic data, we utilized an unsupervised and nonparametric methodology termed the Gene Set Variation Analysis (GSVA) 23 to forecast distinct pathway activities. To identify the pathway associated with immunotherapy, we acquired gene signatures from the study by Hu *et al.* 24. Supplementary Table S1 presents these signatures. The pathway activity scores for the cancer-related pathways were calculated based on the sum of protein expression of all regulatory components in the pathway 25.

### Evaluating immune cell infiltration

To investigate the immune microenvironment, we utilized Cell-type Identification by Estimating Relative Subsets of RNA Transcripts (CIBERSORT) 26, a computational tool that assesses the relative proportions of 22 unique immune cell types infiltrating the tumors in each sample.

### Predicting drug response

The half maximal inhibitory concentration (IC50) of the drugs was estimated utilizing the “pRRophetic” R package 27.

### Small conditional RNA-seq and spatial transcriptome sequencing data analysis

To assess DDIT expression in the tumor microenvironment across various small conditional (sc)RNA-seq datasets, the Tumor Immune Single-cell Hub 28 single cell database was utilized. Furthermore, the SpatialDB database (http://www.spatialomics.org/SpatialDB/) was utilized to evaluate DDIT3 expression in breast cancer through spatial transcriptome sequencing.

For intercellular communication networks, scRNA-seq data with cell cluster annotations and paired bulk RNA-seq data were utilized from 24 breast tumors sourced from the Gene Expression Omnibus (GSE176078) database. The “Seurat R” package 29 was utilized to perform an unsupervised clustering of single cells using the read count matrix as the input. To ensure data quality, the scRNA-seq data underwent quality control measures 30. Additionally, intercellular communication network-related analysis was conducted using the “iTalk R” package 31.

### Statistical analysis

Expression across distinct groups was compared utilizing a one-way analysis of variance and the Student's t-test. A chi-squared test was conducted to investigate the association between DDIT3 expression and baseline clinical features. Spearman's correlation coefficient was calculated to assess the correlation strength and direction. All statistical analyses were performed using SPSS 22.0, SangerBox 32, and R 4.1.3. p≤0.05 indicated statistical significance.

## Result

### DDIT3 expression in breast cancer

**Figure 1:**
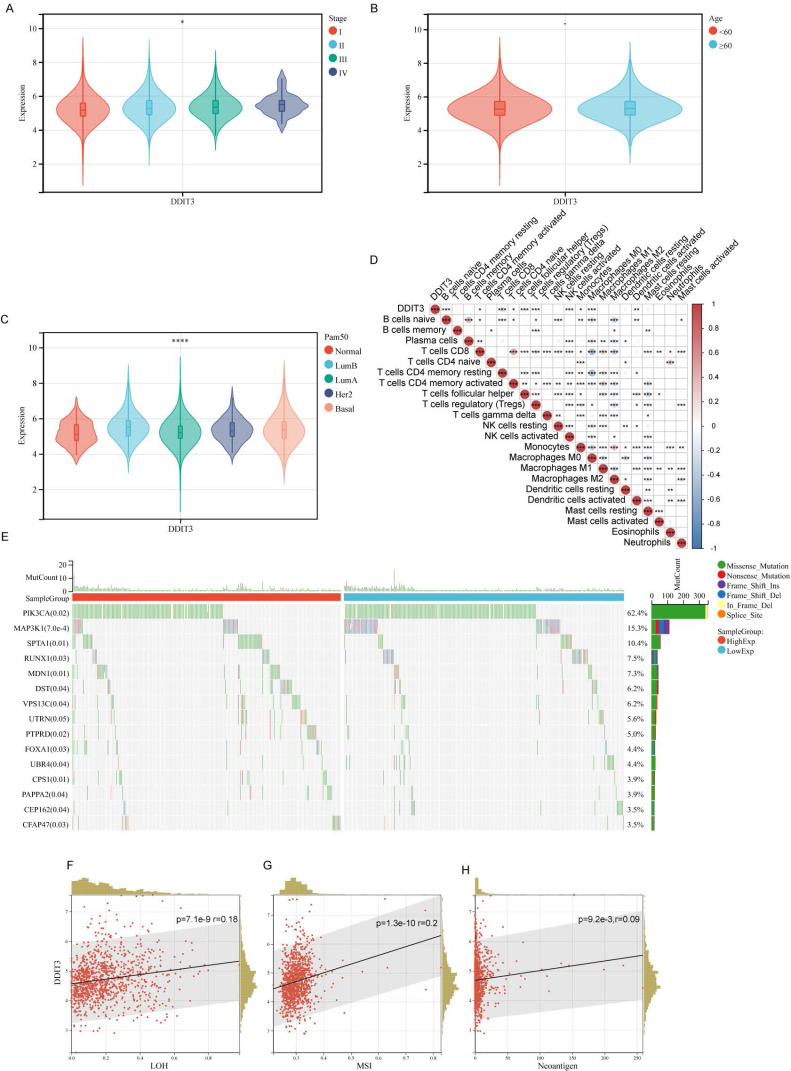
** Association between Clinical Parameters and DDIT3 in Breast Cancer.** (A-C) Transcriptional Profiling of DDIT3 Across Varied Parameters: (A) Stage, (B) Age, and (C) Pam50 Subtype. (D) Exploring the Interplay between DDIT3 Expression and the Composition of 22 Immune Cell Subsets via CIBERSORT Analysis. (E) Deciphering Mutation Landscapes in Cohorts Stratified by high and low DDIT3 Expression. (F-H) Unveiling Correlations between DDIT3 Expression and Parameters encompassing LOH, MSI, and Neoantigen Burden. Statistical Significances are Indicated: *p < 0.05; **p < 0.01; ***p < 0.001; ****p < 0.0001.

We conducted an in-depth investigation using TCGA cohort to explore the association between the clinical attributes in breast cancer and DDIT3. We observed a strong correlation between DDIT3 expression and advancing breast cancer stages (Figure 1A), thereby implying its role in tumor progression. Contrarily, DDIT3 expression did not significantly differ across various age groups (Figure 1B). Delineating the impact across PAM50 subtypes, DDIT3 was characterized by the lowest expression in the Normal subtype and the highest expression in the Luminal B subtype (Figure 1C).

We implemented the CIBERSORT algorithm to explore the correlation between DDIT3 expression and immune cell infiltration. DDIT3 demonstrated a weak positive correlation with CD8+T cells and a robust positive correlation with regulatory T cells (Tregs) (Figure 1D). The genetic landscape was scrutinized for somatic mutations in tandem with DDIT3 expression. We observed abundant SPTA1 mutations and scarce PIK3CA and MAP3K1 mutations within the patient with high expression of DDIT3 (Figure 1E). Moreover, DDIT3 expression was substantially correlated with the loss of heterozygosity, microsatellite instability, and neoantigen formation (Figures 1F-H).

**Figure 2:**
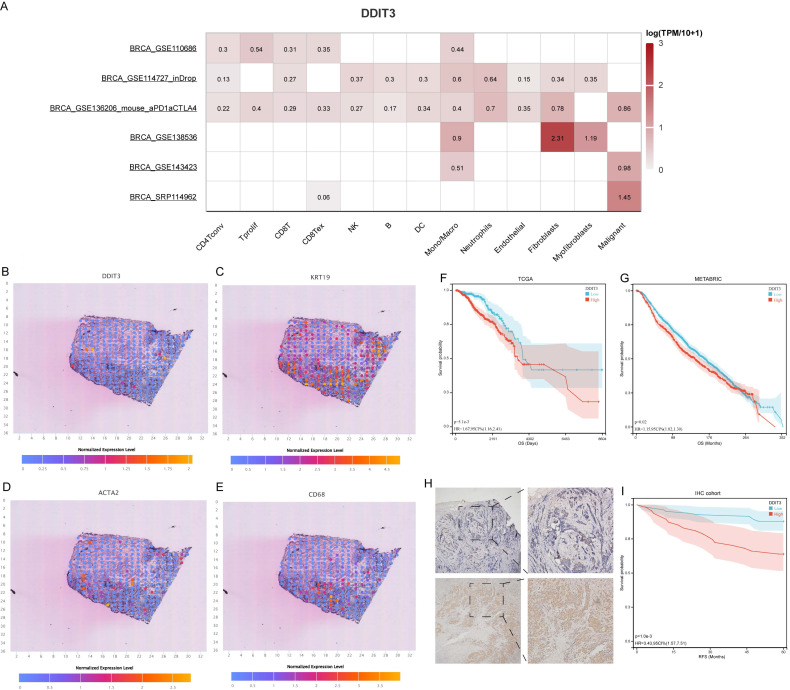
** Expression Pattern and Prognostic Significance of DDIT3 in Breast Cancer.** (A) Differential expression profile of DDIT3 across distinct cellular clusters as derived from diverse scRNA-seq datasets. (B-E) Spatial representation depicting the distribution of expression for (B) DDIT3, (C) KRT19, (D) ACTA2, and (E) CD68 within the context of breast cancer tissue. (F-G) Kaplan-Meier survival curves illustrating the prognostic impact of the DDIT3 subgroup within the (F) TCGA-BRCA cohort and the (G) METABRIC cohort. (H) Exemplary images of immunofluorescence staining portraying cases of low (top) and high (bottom) DDIT3 expression within breast cancer tissue specimens. (I) Kaplan-Meier survival analysis characterizing the DDIT3 within the IHC cohort.

In the subsequent phase, we explored DDIT3 expression across cellular clusters and architectural domains. scRNA data underscored widespread DDIT3 across various cell types within breast cancer. The highest expression was observed in Mono/Macro, fibroblasts, and malignant cells (Figure 2A). This finding was corroborated by spatial transcriptomic sequencing data, which consistently co-localized DDIT3 with the markers of malignancy (KRT19), fibroblasts (ACTA2), and Mono/Macro cells (CD68) (Figures 2B-E). Additionally, a pan-cancer pathway analysis suggested that DDIT3 was positively correlated with apoptosis, cell cycle, DNA damage, and epithelial-mesenchymal transition (EMT) pathways in breast cancer (Figure S1).

Survival analysis through the Kaplan-Meier (KM) methodology elucidated the prognostic implications of DDIT3. Patients with high DDIT3 expression demonstrated an exacerbated prognosis in TCGA cohort (Figure 2F). This finding was consistent within the METABRIC cohort, thus validating the predictive prowess of DDIT3 (Figure 2G). To corroborate these findings, IHC staining was conducted on independent breast cancer samples. DDIT3 was predominantly expressed within the carcinoma cells, with conspicuous localization within both the cellular nucleus and cytoplasmic compartments (Figure 2H). Importantly, the validation analysis accentuated the adverse correlation between elevated DDIT3 expression and recurrence-free survival in patients with breast cancer (Figure 2I).

### Constructing a DDIT3-related prognostic signature in breast cancer

**Figure 3:**
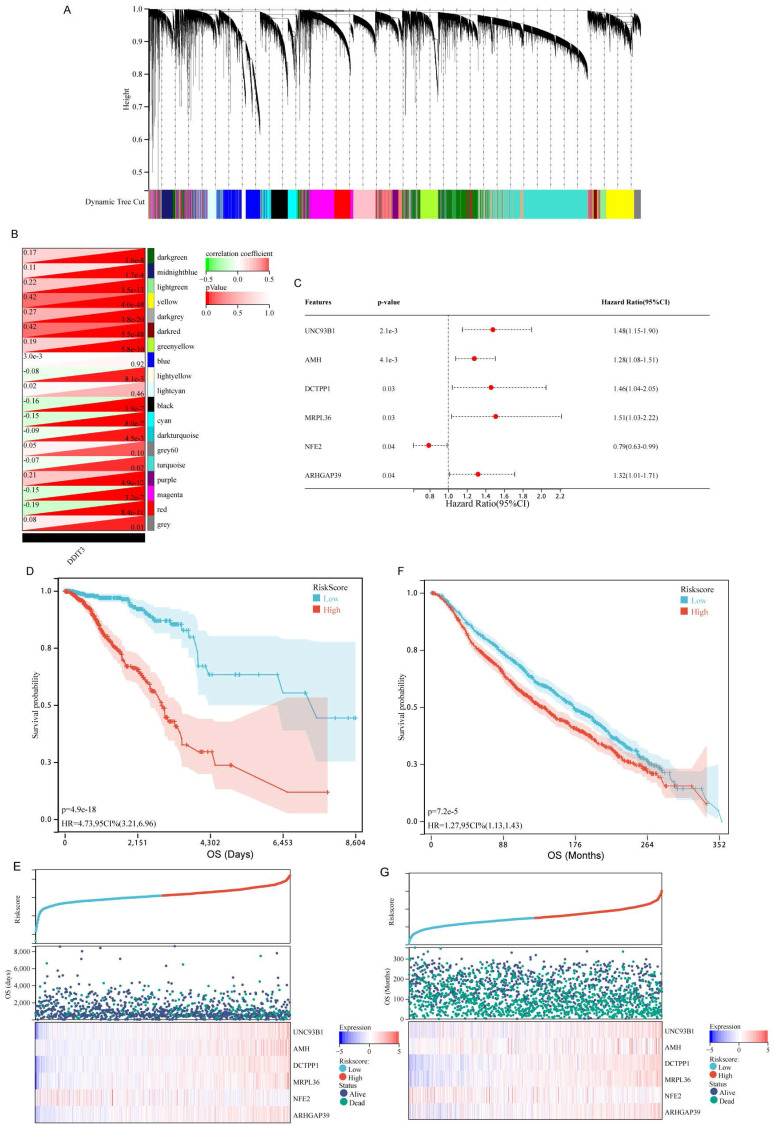
** Discovery of DDIT3-Associated Genes and Development, as well as Validation of a DDIT3-related Prognostic Model in Breast Cancer.** (A-B) (A) Hierarchical agglomerative clustering dendrogram portraying the arrangement of DDIT3-associated genes and (B) heatmap representation depicting the interrelationship among DDIT3-correlated gene modules. (C) Forest plots illustrating multivariate Cox regression analysis utilized for the identification of pivotal genes. (D-E) (D) Kaplan-Meier survival analysis curve and (E) depiction of risk scores along with corresponding survival statuses pertaining to the DDIT3-related prognostic signature in the TCGA cohort. (F-G) (F) Kaplan-Meier survival curve and (G) representation of Risk Scores alongside associated survival statuses with respect to the DDIT3-related prognostic signature in the METABRIC cohort.

To elucidate the prognostic implications of DDIT3-related genes in breast cancer, a comprehensive analysis was conducted utilizing WGCNA. This approach facilitated the identification of 19 distinct gene modules by applying average hierarchical clustering in conjunction with dynamic tree clipping (Figure 3A). The interplay between the pivotal modules and DDIT3 was explored. Of the 19 gene modules, the yellow and dark red modules were strongly correlated with DDIT3 expression (Figure 3B).

Functional annotations demonstrated the distinct roles of the genes within these modules. Specifically, the dark red module gene set was enriched in processes, such as spliceosome assembly (KEGG), mitochondrial gene expression (GO-biological process, GO-BP), spliceosomal tri-snRNP complex constitution (GO-cellular component, CC), and glycolipid binding (GO-molecular function, MF) (Supplementary Figure S2). Similarly, the yellow module was enriched in pathways, such as nicotinate and nicotinamide metabolism (KEGG), mitochondrial electron transport involving nicotinamide adenine dinucleotide (NAD) + hydrogen (H) to ubiquinone (GO-BP), RNA polymerase II activity (GO-CC), and the core complex along with NADH dehydrogenase (quinone) activity (GO-MF) (Supplementary Figure S3).

Subsequently, the genes encapsulated within the dark red and yellow modules were selected for downstream investigations. The univariate Cox regression analysis yielded 138 genes associated with the OS (Supplementary Table S2). To mitigate overfitting, an additional LASSO regression analysis (Supplementary Figure S4A, B) and multivariate Cox regression analysis (Figure 3C) were conducted.

Finally, we identified a robust DDIT3-associated prognostic signature, including six pivotal genes, namely, unc-93 homolog B1, TLR signaling regulator (UNC93B1), anti-Mullerian hormone (AMH), DCTP pyrophosphatase 1 (DCTPP1), mitochondrial ribosomal protein L36 (MRPL36), nuclear factor erythroid 2 (NFE2), and Rho GTPase activating protein 39 (ARHGAP39). The risk score was calculated as follows: Risk score = 0.390644393 * UNC93B1 + 0.243192629 * AMH + 0.38007083 * DCTPP1 + 0.414956757 * MRPL36 - 0.24076553 * NFE2 + 0.276104403 * ARHGAP39. It facilitated characterizing the prognostic stratification of patients.

The predictive potency of this risk score was validated through the KM survival analyses. Notably, patients within the high-risk group demonstrated markedly diminished survival probabilities within TCGA cohort (Figures 3D and 3E). This trend was consistent in the KM survival curves derived from the METABRIC cohort, reinforcing the reliability of our findings across distinct patient populations (Figures 3F and 3G).

### Functional annotations and landscape of the somatic mutations of DDIT3-related risk score-based classification

**Figure 4:**
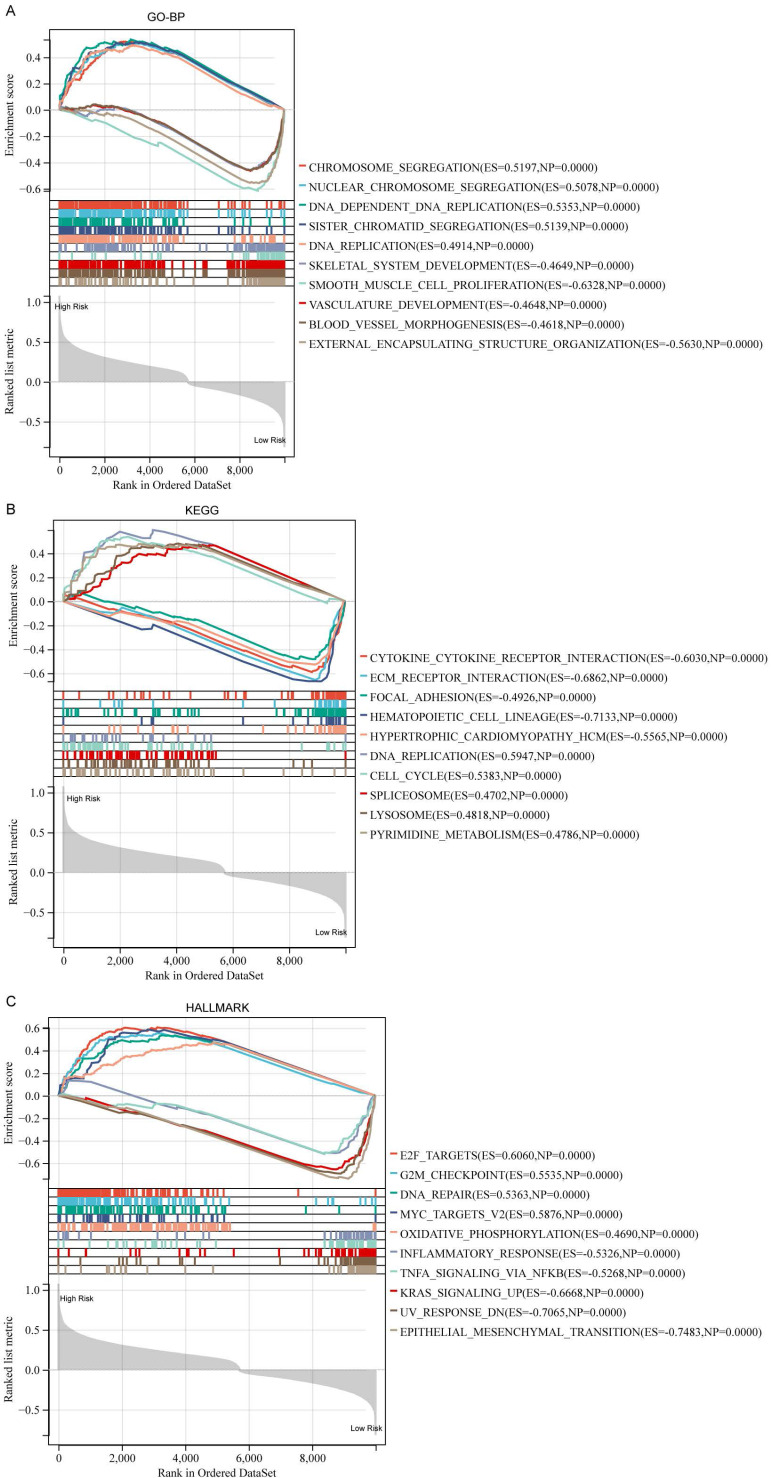
** Functional analysis for DDIT3-related risk score in breast cancer.** Functional scrutiny of the DDIT3-related risk score through GSEA predicated on gene sets sourced from (A) GO-BP, (B) KEGG, and (C) Hallmark gene sets.

To understand the mechanisms underlying the prognostic implication of the DDIT3-related risk score, a comprehensive GSEA was undertaken. Patients with breast cancer classified into the high-risk group demonstrated substantially enriched processes encompassing chromosome segregation, nuclear chromosome segregation, and DNA-dependent DNA replication within the GO-BP framework (Figure 4A). Similarly, this high-risk cohort demonstrated considerably enriched DNA replication, cell cycle regulation, and spliceosome assembly within the KEGG pathway database (Figure 4B). These enriched signatures extended to the key biological processes, such as E2F-targeted pathways, G2M checkpoint regulation, and DNA repair mechanisms, as evident in the Hallmark gene set (Figure 4C).

Conversely, patients classified into the low-risk group demonstrated distinct enrichment signatures. In the GO-BP domain, they demonstrated enriched processes, including smooth muscle cell proliferation, external encapsulating structure organization, and skeletal system development (Figure 4A). A similar pattern emerged in the KEGG pathway analysis, with considerably enriched hematopoietic cell lineage, ECM receptor interaction, and cytokine-cytokine receptor interactions (Figure 4B). Notably, the Hallmark gene set analysis unveiled enriched pathways, such as epithelial-mesenchymal transition, ultraviolet response DNA damage, and Kristen Rat Sarcoma Viral oncogene homolog signaling upregulation for the low-risk cohort (Figure 4C).

Furthermore, we observed discernible patterns in the mutational landscape of patients within the high- and low-risk groups. Patients in the high-risk group demonstrated a higher mutation frequency in the TP53, GATA3, and Duchenne muscular dystrophy genes. By contrast, patients in the low-risk group demonstrated a higher mutation frequency in the MUC16 and SYNE2 genes (Supplementary Figure S5). This mutational profile accentuated the potential mechanistic disparities underlying the prognostic outcomes associated with the DDIT3-related risk score.

### Tumor immune microenvironment features based on the DDIT3-related prognostic signature

**Figure 5:**
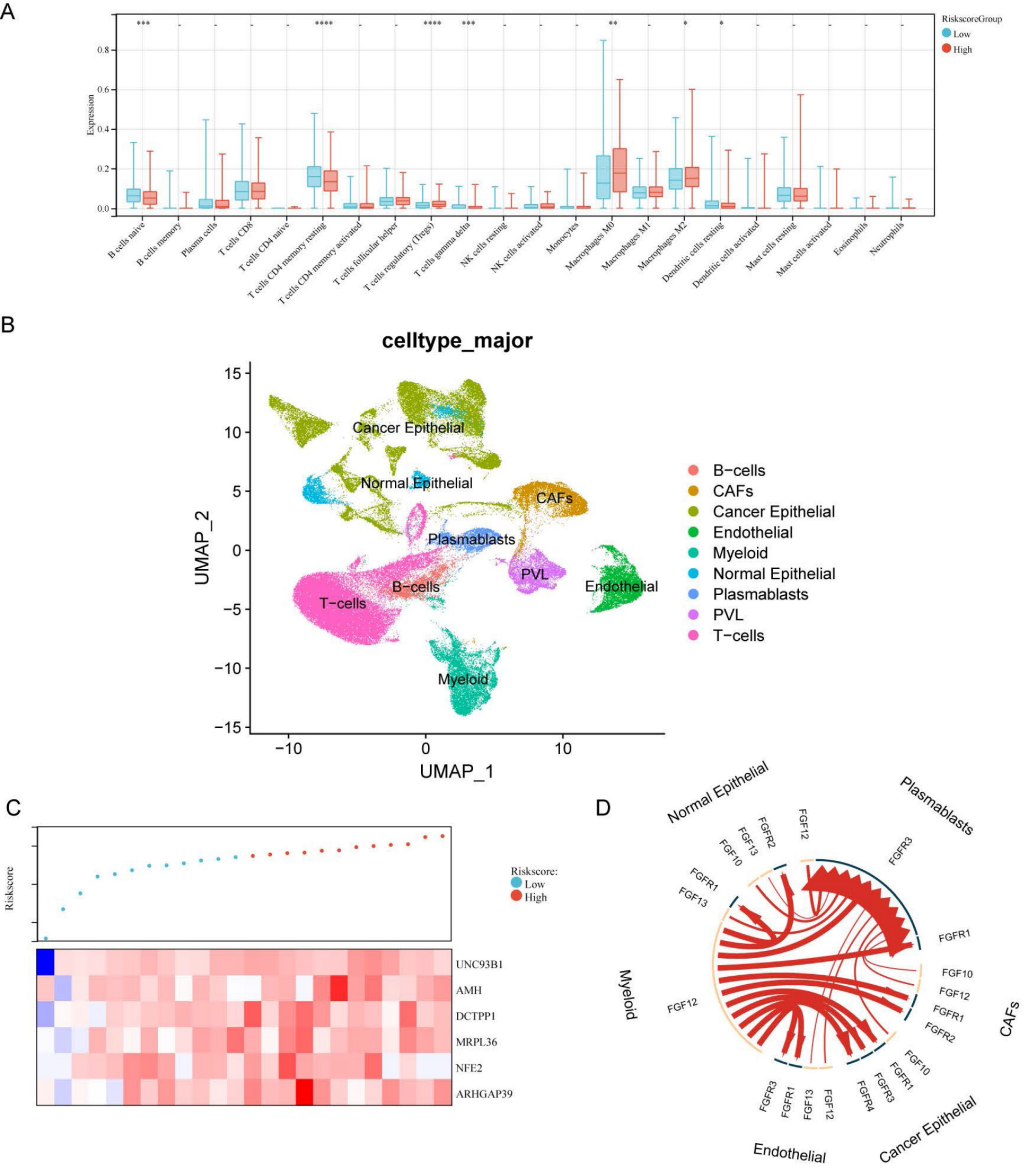
** Correlation Assessment Between DDIT3-related Risk Score and Tumor Immune Microenvironment.** (A) Estimation of the relative abundance of 22 distinct immune cell types within high and low-risk groups of breast cancer utilizing the CIBERSORT algorithm. (B) UMAP visualization portraying the principal subpopulations of cells within the GSE176078 cohort. (C) Hierarchical ordering of risk scores predicated on bulk RNA-seq expression within the GSE176078 cohort., (D) Circular diagram displaying the 20 most notably perturbed differential receptor-ligand interactions between the high-risk and low-risk cohorts, with heightened expression in the high-risk group denoted by the red coloration. *p < 0.05; **p < 0.01; ***p < 0.001; ****p < 0.0001.

We explored the intricate correlation between the infiltration patterns of tumor-infiltrating immune cells and the risk score. The patients in the high-risk category demonstrated increased infiltration levels of Tregs, macrophages of M0 phenotype, and macrophages of M2 phenotype and decreased levels of naïve B cells, resting memory CD4+ T cells, and gamma delta T cells (Figure 5A). Collectively, these results implicate an immunosuppressive tumor immune microenvironment (TIME) in high-risk breast cancer cases.

A dataset encompassing scRNA- and paired bulk RNA-seq data from 24 patients with breast cancer was acquired from GSE176078 to explore the functional implications of the risk score within the TIME (Figure 5B). In line with the risk score stratification, these patients were classified into high- and low-risk groups. The median risk score served as the demarcation point (Figure 5C). We utilized iTalk, a computational framework to decipher intercellular interactions grounded in ligand-receptor signal transduction. Specifically, the 20 most significantly altered receptor-ligand pairs were elevated in the high-risk group. Notably, these interactions were attributed to members of the fibroblast growth factor (FGF) and FGF receptor (FGFR) family (Figure 5D). This insight elucidates the pivotal role of FGF-FGFR interactions in mediating the interplay within the high-risk breast cancer microenvironment.

### DDIT3-related prognostic signature predicts immunotherapy opportunities

**Figure 6:**
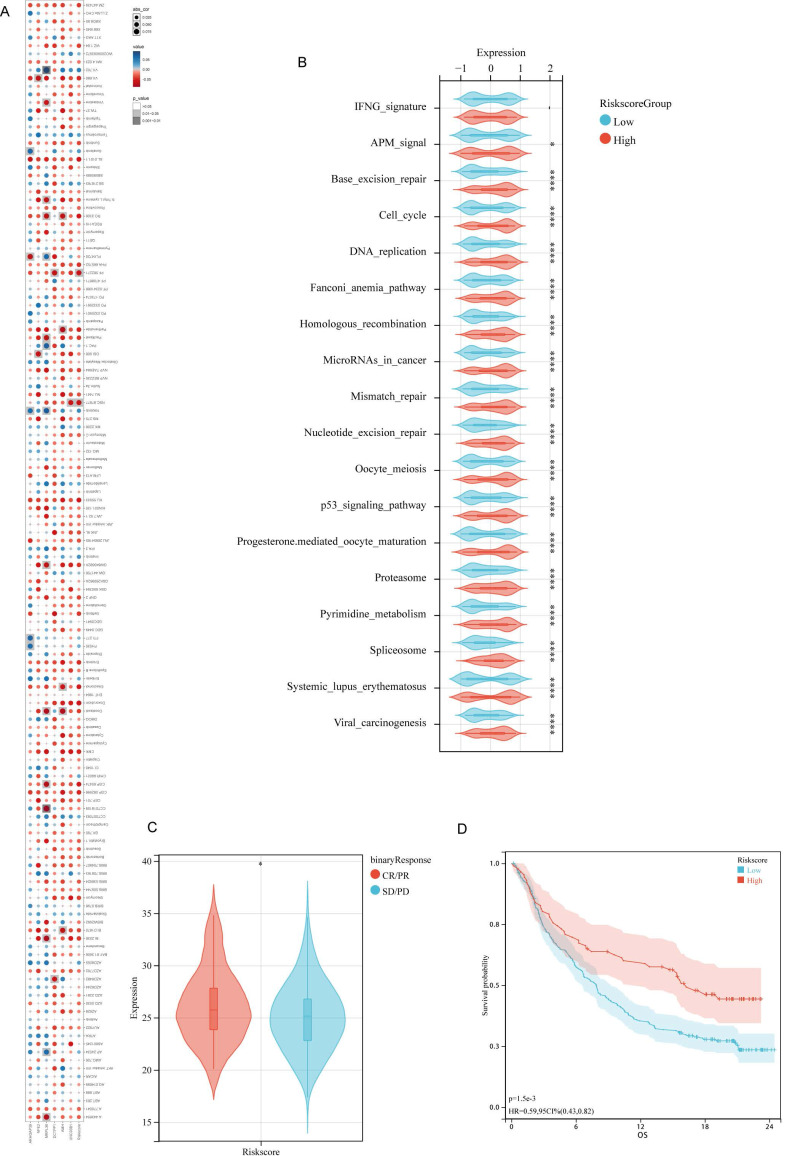
** Correlative Investigation of DDIT3-related Risk Score with Drug Sensitivity and Immunotherapy Responsiveness.** (A) Examination of the interrelationship among IC50 values of diverse drugs, risk score, and model genes. (B) Differential expression of enrichment scores related to pathways pertinent to immunotherapy within high and low-risk group. (C) Evaluation of risk score distribution among patients demonstrating different clinical responses to cancer immunotherapy within the IMvigor210 cohort. (D) Kaplan-Meier survival analysis delineating the prognostic significance of the risk score within the IMvigor210 cohort. *p < 0.05; **p < 0.01; ***p < 0.001; ****p < 0.0001.

To explore the interplay between the risk score and drug sensitivity, we assessed the IC50 values for each drug pertinent to breast cancer within TCGA cohort. Intriguingly, of 138 drugs, only two drugs, namely NSC.87877 and PF.562271, demonstrated a substantial correlation between their sensitivity profiles and the risk score (Figure 6A). Notably, their key targets, namely SHP-2 (targeted by NSC.87877) and FAK (targeted by PF.562271), are the downstream effectors within the FGF pathway 33,34. Therefore, the FGF/FGFR signaling cascade is central to the high-risk patient subgroup.

Considering the distinct TIME characteristics of immunosuppression in high-risk patients, we explored the differences in predictive immunotherapy pathways between the high- and low-risk subgroups within TCGA-BRCA cohort. All immunotherapy prediction pathway scores were significantly upregulated in the high-risk patient group (Figure 6B). Sequencing data were unavailable from patients with breast cancer receiving immunotherapy. Therefore, we utilized the IMvigor210 cohort for bladder cancer immunotherapy to validate the predictive power of the prognostic models on the efficacy of immunotherapy. This cohort comprises patients with cancer treated with anti-PD-L1 therapy. Moreover, it possesses comprehensive RNA-seq data along with clinical follow-up. It has been widely utilized in prognostic models to predict the efficacy of immunotherapy 35-38. Patients with higher risk scores in the IMvigor210 cohort were more likely to respond to immunotherapy (Figure 6C), thereby achieving a favorable prognosis. This comparison highlights the potential predictive capability of the risk scores in identifying responses to immunotherapy and subsequent clinical outcomes (Figure 6D).

### Constructing a nomogram to predict breast cancer survival

**Figure 7:**
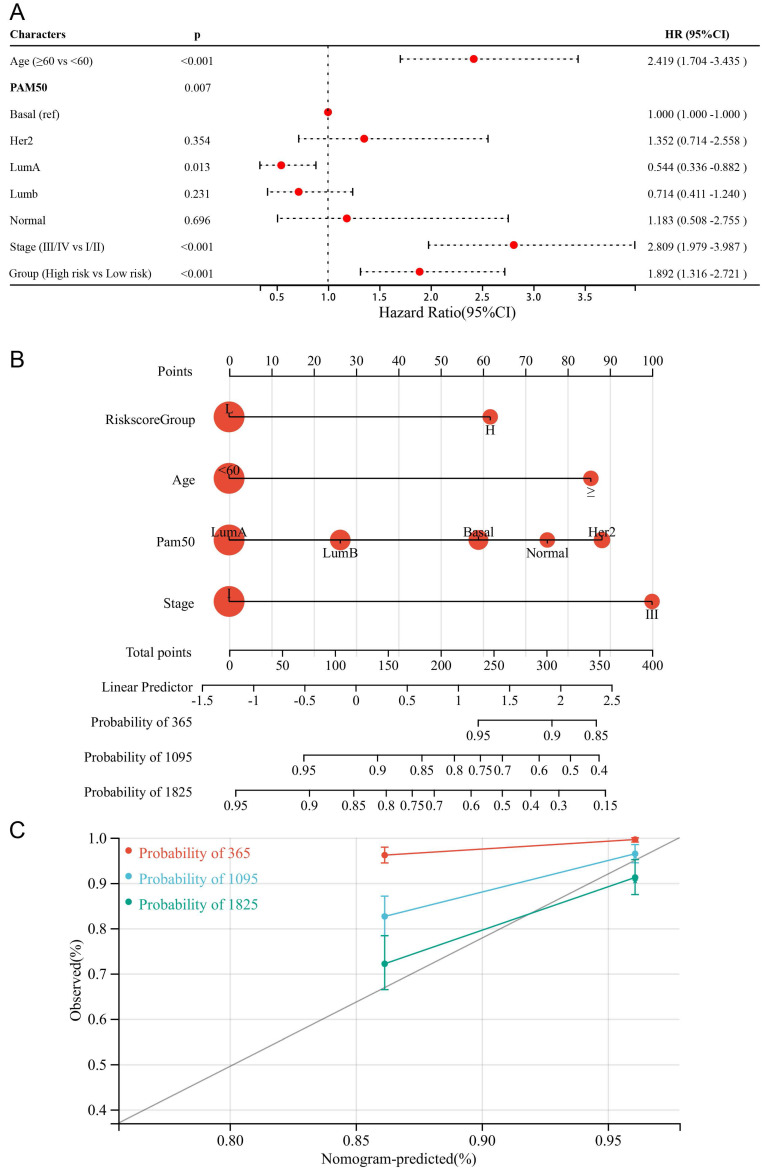
** Formulation and Evaluation of the Nomogram Survival Model.** (A) Display of forest plots illustrating the outcomes of multivariate regression integrating clinical factors and DDIT3-related risk score. (B) Nomogram representation for the prediction of overall survival probability, with age, Pam50 subtype, risk score group, and tumor stage being utilized as predictive parameters. (C) Calibration curves confirming the accuracy of predictions; red denotes 1-year predictions, blue signifies 3-year predictions, and green represents 5-year predictions.

To make the DDIT3-related prognostic signature more clinically applicable, we conducted multivariate Cox regression analysis to select the most suited variables for inclusion within the forest plot (Figure 7A). The variables of age, tumor stage, and risk score grouping were identified as the potential candidates to function as independent prognostic determinants. Subsequently, we formulated a pioneering predictive nomogram, including age, tumor stage, and risk score group as the pivotal parameters (Figure 7B). The calibration curves rigorously evaluated the precision of this nomogram, highlighting its ability to accurately predict the survival probabilities across diverse scenarios (Figure 7C). This innovative tool can aid clinical practitioners in prognostic assessments and informed decision-making for patients with breast cancer.

## Discussion

Our comprehensive investigation into the immunological effects and prognostic implications of DDIT3 in breast carcinoma has indicated its diverse functions and potential clinical application. Unlike previous studies 11,39, we utilized an integrative bioinformatics approach coupled with IHC, which has not been extensively explored regarding the role of DDIT3 in breast cancer. Prior research has demonstrated the association between DDIT3 and cellular mechanisms. However, we established a correlation between DDIT3 and adverse outcomes in patients with breast cancer. We introduced a novel prognostic signature associated with DDIT3 expression, immune microenvironment modulation, and response to therapy in breast cancer. We conducted a broad spectrum of analyses, including transcriptional profiling, clinical correlations, immune interactions, mutational landscapes, and therapeutic insights.

The biological functionality attributed to DDIT3 resides in its role as a transcription factor within the cellular stress response pathways. Serving as a pivotal orchestrator, DDIT3 mediates the coordination of transcriptional programs pivotal to preserving cellular homeostasis during adverse circumstances, such as ER stress, nutrient insufficiency, and oxidative stress 3.

Notably, the transcriptional activity of DDIT3 precipitates gene modulation that mediates protein folding, the unfolded protein response (UPR), apoptosis, and lipid metabolism, thus ensuring cellular adaptation and endurance under adverse conditions 4. In oncology, the dualistic involvement of DDIT3 has gained considerable attention. DDIT3 functions as a sentinel against advancing tumors by initiating apoptosis and arresting proliferation. Nonetheless, its aberrant activation can potentiate oncogenesis by nurturing tumor survival, angiogenesis, and metastasis 12,13,40. Furthermore, DDIT3 expression is significantly upregulated in gastric cancer. Moreover, it promotes the stemness of cancer stem cells in gastric cancer by regulating CCAAT enhancer-binding proteins β 41. The functional repertoire of DDIT3 is characterized by a striking sensitivity to the cellular context, which is closely associated with the distinct TME features and state. The roles of DDIT3 in human cancers are shaped by various factors, namely cancer-specific genetic mutations, the temporal evolution of diseases, and elaborate crosstalk with signaling networks 3. These subtle distinctions emphasize the complexity of its biological role, underscoring the importance of considering the distinctive properties of each cancer entity when dissecting their contributions to cancer biology. With an increased understanding of the molecular interplay governed by DDIT3, targeting its functions appears promising for developing therapeutic strategies against malignancies.

DDIT3 expression pattern in breast cancer is associated with the clinical parameters. The expression increases with advanced stages, suggesting its involvement in tumor progression. Varied expressions across different PAM50 molecular subtypes suggest subtype-specific roles. These findings highlight the contribution of DDIT3 to the heterogeneity of breast cancer and its potential utility as a diagnostic and prognostic biomarker. Additionally, the correlations between DDIT3 expression and immune cell infiltration patterns imply its plausible role in shaping the tumor microenvironment and modulating immune responses. The somatic mutational landscape associated with DDIT3 expression underscores potential molecular mechanisms. Associations with SPTA1, PIK3CA, and MAP3K1 mutations suggest the role of DDIT3 in diverse pathways in cancer progression. Furthermore, the correlation between DDIT3 expression and the loss of heterozygosity, microsatellite instability, and neoantigen burden underscores its potential effect on genomic instability inherent to breast cancer. Furthermore, analysis of the oncogenic pathways highlighted the role of DDIT3 in cellular processes, including apoptosis, the cell cycle, DNA damage response, and EMT. In breast cancer, high DDIT3 expression is positively correlated with these pathways, reflecting the biological response of tumor cells to ER stress. ER stress can cause the accumulation of misfolded proteins, thereby activating the UPR. DDIT3 responds to chronic ER stress by inhibiting cell cycle progression and promoting molecules involved in the apoptosis pathways 3. Additionally, DDIT3 is involved in regulating DNA repair-related gene expression, affecting the response to DNA damage 42. The role of DDIT3 in the EMT process may be attributed to modulating the expression of adhesive molecules and matrix metalloproteinases, which are involved in tumor cell invasion and metastasis 43. Consequently, high DDIT3 expression may be associated with increased invasiveness and metastatic potential in breast cancer, which are correlated with poorer prognosis.

Exploring DDIT3 expression within cellular clusters and architectural contexts improved our understanding of its spatial distribution. High expression within Mono/Macro cells, fibroblasts, and malignant cells underscores its intricate role in modulating the tumor microenvironment and cellular communication. This observation was confirmed by the spatial transcriptome sequencing data, which elucidated DDIT3 co-localization with the markers indicating diverse cellular populations, contributing to the spatial context of its functions. Researchers have reported an intricate interaction network, wherein DDIT3 regulates a complex interplay of molecular responses within the non-neoplastic cells inhabiting the tumor niche 15,44. DDIT3 is overexpressed within myeloid-derived suppressor cells; moreover, it participates in the functional modulation of the transplanted CD8+ T cells. Furthermore, the tumor growth rate is notably reduced in DDIT3-deficient mice 15. This research highlights the potential of DDIT3 as a pivotal node for therapeutic interventions targeting the interplay between the tumor microenvironment and its non-neoplastic inhabitants in breast cancer. The role of DDIT3 in regulating cellular stress responses and apoptosis as well as its complex interactions within the tumor microenvironment suggests it as a promising therapeutic target. Modulating DDIT3 activity may lead to the development of novel treatment strategies that inhibit tumor growth and metastasis. However, considering the multifunctionality of DDIT3 and its sensitivity to the cellular environment, future treatment strategies will necessitate a personalized approach for the patients.

The DDIT3-associated prognostic signature served as a potential tool for classifying the patients and predicting prognosis. Utilizing methodologies, such as WGCNA, univariate, LASSO, and multivariate Cox regression analyses, we formulated a robust prognostic signature encompassing six genes, namely UNC93B1, AMH, DCTPP1, MRPL36, NFE2, and ARHGAP39. Some of these genes have been implicated in breast cancer 45,46, whereas others remain less explored. For example, UNC93B1 promotes tumoral growth by regulating granulocyte-macrophage colony-stimulating factor secretion in human oral cancer; nonetheless, it has not yet been associated with breast cancer 47. Elevated serum levels of AMH have been associated with increased risk and adverse prognosis in breast cancer. However, the impact of intrinsic AMH expression in breast cancer cells on their tumoral biology remains unexplored 48-50. MRPL36, a mitochondrial ribosomal protein, warrants investigation in BRCA. Nonetheless, it has been correlated with poor progression-free survival in ovarian cancer 51. ARHGAP39, also termed preoptic regulatory factor-2 or Vilse belongs to the GTPase activating protein group. It is central to neurogenesis and neurodevelopment. However, its role in cancer remains unclear 52.

We investigated the functional annotations associated with the risk score-based classification for DDIT3, highlighting the intricate biological processes underlying its prognostic implication. The increased prevalence of pathways related to DNA replication, cell cycle regulation, and DNA repair within the high-risk cohort accentuated prospective mechanisms regulating aggressive disease behavior. By contrast, the enriched pathways correlated with the proliferated immune cells among the low-risk group suggested a milieu characterized by decreased aggressiveness alongside increased immune activity. Particularly, the DDIT3-related risk score highlighted compelling associations with therapeutic prospects. Notably, we identified increased responsiveness to specific therapeutic agents, such as NSC.87877 and PF.562271, indicating potential targets for intervention. This finding is important because it pertains to the FGF/FGFR pathway, a participant in cancer advancement and immune modulation 53,54. Furthermore, the effects of the DDIT3-associated prognostic signature hold relevance in immunotherapy response. The increased expression of immunotherapy-related pathways within the high-risk group suggests plausible avenues for precision-oriented immunotherapeutic interventions.

Taken together, a nomogram that included age, tumor stage, and risk score as the independent prognostic determinants has emerged as a tool for predicting outcomes in clinical settings. Its precision in predicting survival probabilities highlights its potential utility for informed clinical decision-making. Nevertheless, this study had some limitations. First, its retrospective design warrants validation through prospective clinical trials encompassing larger patient cohorts. Second, the data analysis was based on only digital repositories and clinical specimens, warranting external validation through *in vitro* and *in vivo* experimentation and trials.

## Conclusion

This study demonstrated the implications of DDIT3 in breast cancer, suggesting its role in disease progression, immune modulation, mutational landscapes, and potential treatment. Our findings improved our understanding of the role of DDIT3 in breast cancer and suggested promising applications in clinical practice.

## Supplementary Material

Supplementary figures and tables.

## Figures and Tables

**Table 1:** Clinical information of patients in TCGA-BRCA cohort. Bold indicates p-values less than 0.05.

Characteristics	High(N=545)	Low(N=546)	Total(N=1091)	p value
**Age**				0.42
<60	282(25.85%)	297(27.22%)	579(53.07%)	
≥60	262(24.01%)	249(22.82%)	511(46.84%)	
NA	1(0.09%)	0(0.0e+0%)	1(0.09%)	
**Pam50**				**2.9e-4**
LumA	252(23.10%)	312(28.60%)	564(51.70%)	
LumB	133(12.19%)	82(7.52%)	215(19.71%)	
Her2	45(4.12%)	37(3.39%)	82(7.52%)	
Basal	99(9.07%)	91(8.34%)	190(17.42%)	
Normal	16(1.47%)	24(2.20%)	40(3.67%)	
**Stage**				0.14
I	78(7.15%)	103(9.44%)	181(16.59%)	
II	310(28.41%)	309(28.32%)	619(56.74%)	
III	136(12.47%)	111(10.17%)	247(22.64%)	
IV	11(1.01%)	9(0.82%)	20(1.83%)	
NA	10(0.92%)	14(1.28%)	24(2.20%)	

**Table 2:** Clinical information of patients in METABRIC cohort. Bold indicates p-values less than 0.05.

Characteristics	High(N=952)	Low(N=952)	Total(N=1904)	p value
**Age**				0.68
<60	426(22.37%)	416(21.85%)	842(44.22%)	
≥60	526(27.63%)	536(28.15%)	1062(55.78%)	
**Pam50**				**1.0e-7**
LumA	251(13.18%)	347(18.22%)	598(31.41%)	
LumB	375(19.70%)	389(20.43%)	764(40.13%)	
Her2	145(7.62%)	99(5.20%)	244(12.82%)	
Basal	149(7.83%)	95(4.99%)	244(12.82%)	
Normal	32(1.68%)	22(1.16%)	54(2.84%)	
**Stage**				0.10
I	226(11.87%)	249(13.08%)	475(24.95%)	
II	403(21.17%)	397(20.85%)	800(42.02%)	
III	68(3.57%)	47(2.47%)	115(6.04%)	
IV	2(0.11%)	7(0.37%)	9(0.47%)	
NA	253(13.29%)	252(13.24%)	505(26.52%)	

**Table 3:** Clinical information of patients in IHC cohort. Bold indicates p-values less than 0.05.

Characteristics	High(N=64)	Low(N=65)	Total(N=129)	p value
**Age**				0.62
<60	51(39.53%)	55(42.64%)	106(82.17%)	
≥60	13(10.08%)	10(7.75%)	23(17.83%)	
**Molecular Subtypes**				0.50
LumA	16(12.40%)	16(12.40%)	32(24.81%)	
LumB	26(20.16%)	20(15.50%)	46(35.66%)	
Her2	6(4.65%)	11(8.53%)	17(13.18%)	
Basal	16(12.40%)	18(13.95%)	34(26.36%)	
**Stage**				0.81
I	16(12.40%)	20(15.50%)	36(27.91%)	
II	22(17.05%)	22(17.05%)	44(34.11%)	
III	21(16.28%)	20(15.50%)	41(31.78%)	
IV	5(3.88%)	3(2.33%)	8(6.20%)	

## Abbreviations

AMH: anti-Mullerian hormone
ARHGAP39: Rho GTPase activating protein 39
CEBPβ: CCAAT enhancer binding proteins β
CHOP: CCAAT/enhancer-binding protein homologous protein
CIBERSORT: cell-type identification by estimating relative subsets of RNA transcripts
DCTPP1: DCTP pyrophosphatase 1
DDIT3: DNA damage-inducible transcript 3
FGF: fibroblast growth factor
FGFR: fibroblast growth factor receptor
GO: Gene Ontology
GSEA: Gene Set Enrichment Analysis
GSVA: Gene Set Variation Analysis
GO-BP: GO-Biological Process
CC: GO-Cellular Component
MF: GO-Molecular Function
IC50: half maximal inhibitory concentration
HRP: horseradish peroxidase
IHC: Immunohistochemical
KEGG: Kyoto Encyclopedia of Genes and Genomes
METABRIC: Molecular Taxonomy of Breast Cancer International Consortium
MAF: Mutation Annotation Format
MRPL36: mitochondrial ribosomal protein L36
NFE2: nuclear factor erythroid 2
OS: overall survival
Porf-2: preoptic regulatory factor-2
RFS: recurrence-free survival
RNA-seq: RNA sequencing
Tregs: T cells regulatory
TCGA: The Cancer Genome Atlas
TIME: tumor immune microenvironment
TISCH: Tumor Immune Single-cell Hub
UPR: unfolded protein response
WGCNA: Weighted Gene Co-expression Network Analysis
