# Supplementary material for: DDIT3 is associated with breast cancer prognosis and immune microenvironment: an integrative bioinformatic and immunohistochemical analysis
Source: J Cancer. 2024 May 28;15(12):3873–89. doi: 10.7150/jca.96491 (PMC11190778; doi:10.7150/jca.96491)
Supplement: Supplementary file 1 — Supplementary figures and tables. [file jcav15p3873s1.pdf]

Supplementary Figure S1 Bubble plot of the correlation between pan cancer carcinogenic pathway and DDIT3 expression.

Supplementary Figure S2 Functional analysis for the darkred module genes

Functional analysis of genes residing within the darkred module based on gene sets using (A) KEGG, (B) GO-BP, (C) GO-CC, and (D) GO-MF.

Supplementary Figure S3 Functional analysis for the yellow module genes

Functional analysis of genes residing within the yellow module based on gene sets using (A) KEGG, (B) GO-BP, (C) GO-CC, and (D) GO-MF.

Supplementary Figure S4 Screening of DDIT3-related genes in breast cancer.

(A) Coefficient profiles as obtained from the LASSO regression model. (B) Cross-validation process employed for the selection of tuning parameters within the LASSO regression model.

Supplementary Figure S5 DDIT3-related risk score and mutation landscape.

Comparative analysis of the mutation landscape between cohorts classified as having high and low risk scores.

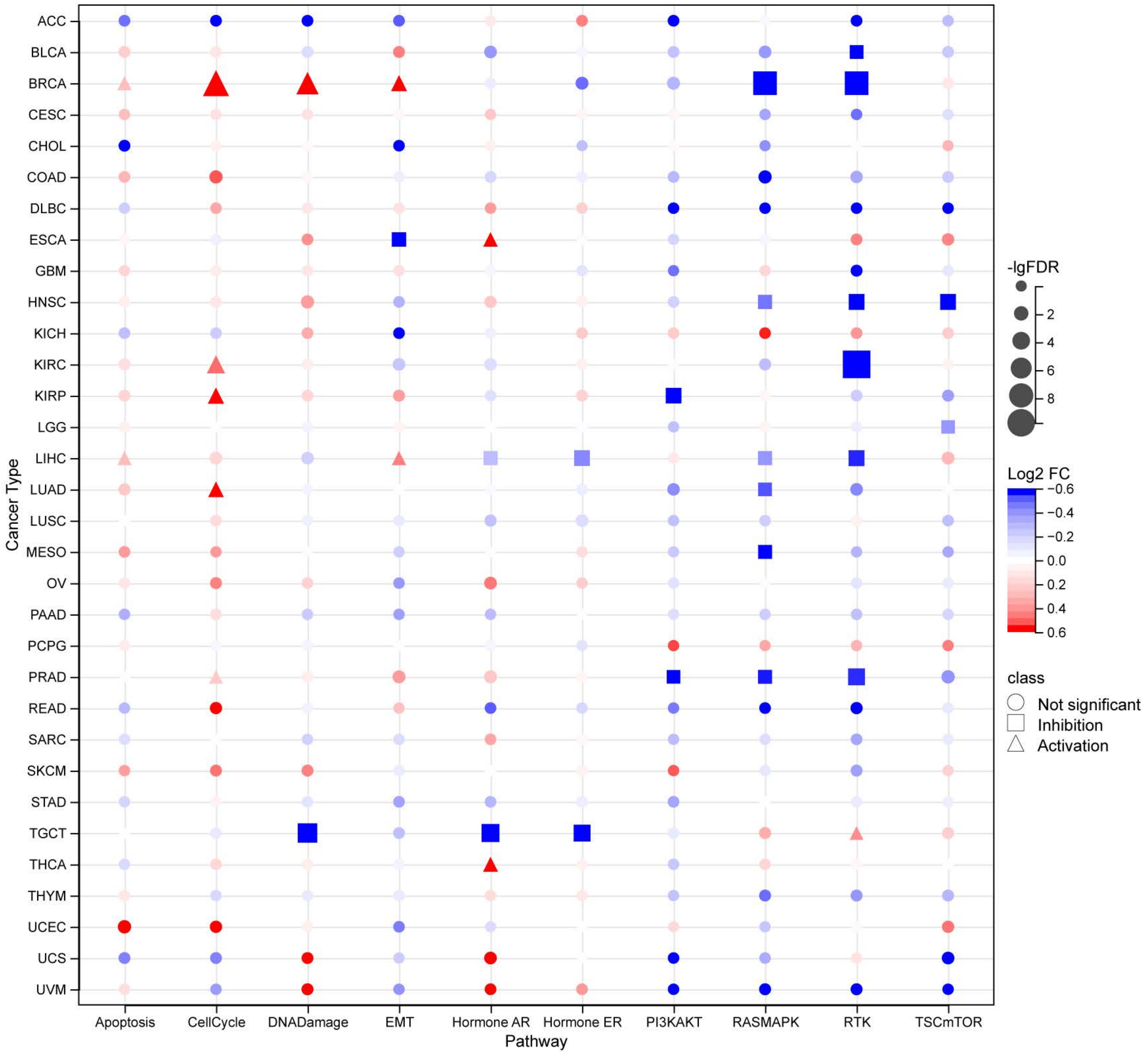

ACC
BLCA
BRCA
CESC
CHOL
COAD
DLBC
ESCA
GBM
HNSC
KICH
KIRC
KIRP
LGG
LIHC
LUAD
LUSC
MESO
OV
PAAD
PCPG
PRAD
READ
SARC
SKCM
STAD
TGCT
THCA
THYM
UCEC
UCS
UVM
Cancer Type
Apoptosis
CellCycle
DNADamage
EMT
Hormone AR
Hormone ER
PI3KAKT
RASMAPK
RTK
TSCmTOR
Pathway
-lgFDR
2
4
6
8
Log2 FC
−0.6
−0.4
−0.2
0.0
0.2
0.4
0.6
class
Not significant
Inhibition
Activation

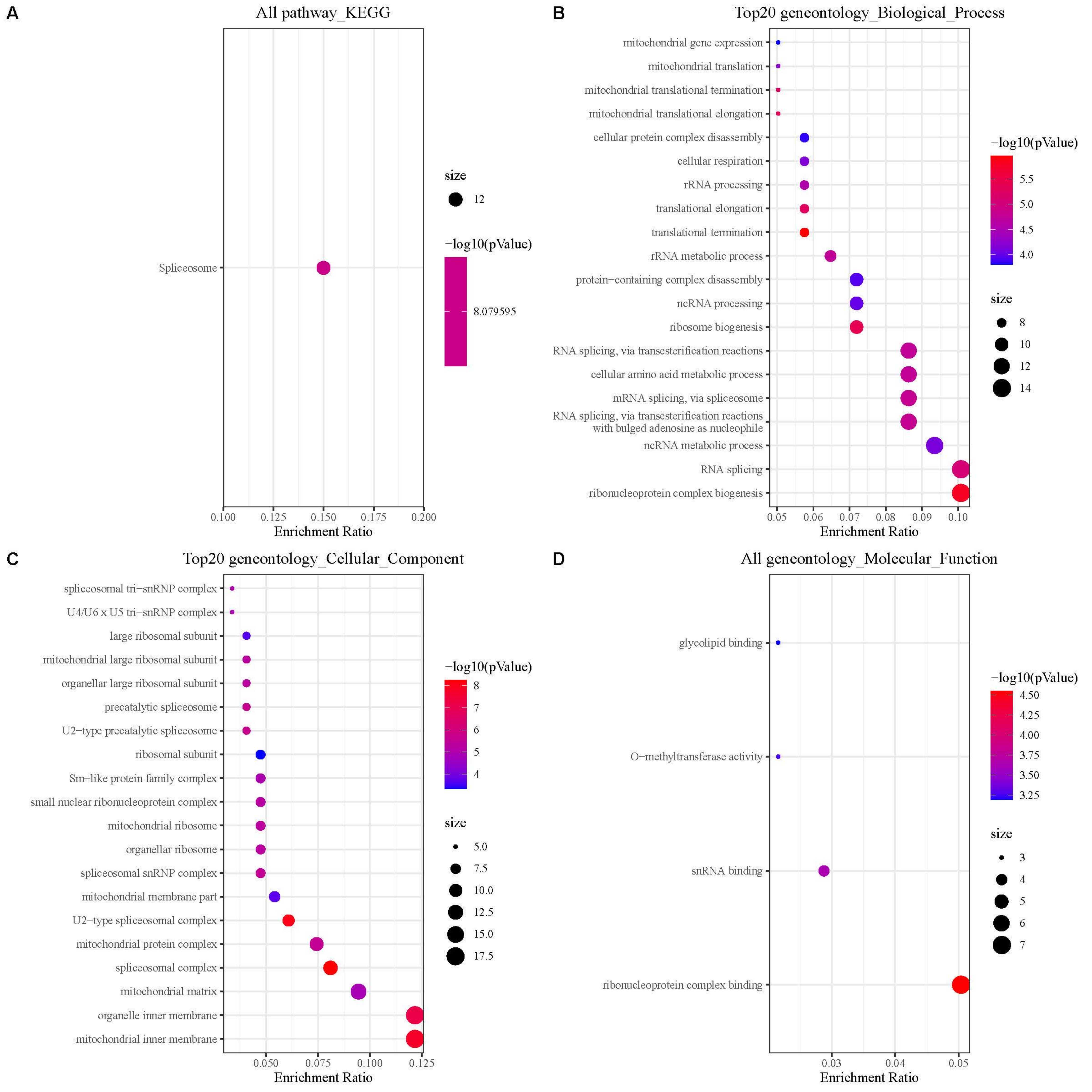
A
All pathway_KEGG
Spliceosome
size
12
−log10(pValue)
8.079595
0.100
0.125
0.150
0.175
0.200
Enrichment Ratio
B
Top20 geneontology_Biological_Process
mitochondrial gene expression
mitochondrial translation
mitochondrial translational termination
mitochondrial translational elongation
cellular protein complex disassembly
cellular respiration
rRNA processing
translational elongation
translational termination
rRNA metabolic process
protein−containing complex disassembly
ncRNA processing
ribosome biogenesis
RNA splicing, via transesterification reactions
cellular amino acid metabolic process
mRNA splicing, via spliceosome
RNA splicing, via transesterification reactions with bulged adenosine as nucleophile
ncRNA metabolic process
RNA splicing
ribonucleoprotein complex biogenesis
−log10(pValue)
5.5
5.0
4.5
4.0
size
8
10
12
14
0.05
0.06
0.07
0.08
0.09
0.10
Enrichment Ratio
C
Top20 geneontology_Cellular_Component
spliceosomal tri−snRNP complex
U4/U6 x U5 tri−snRNP complex
large ribosomal subunit
mitochondrial large ribosomal subunit
organellar large ribosomal subunit
precatalytic spliceosome
U2−type precatalytic spliceosome
ribosomal subunit
Sm−like protein family complex
small nuclear ribonucleoprotein complex
mitochondrial ribosome
organellar ribosome
spliceosomal snRNP complex
mitochondrial membrane part
U2−type spliceosomal complex
mitochondrial protein complex
spliceosomal complex
mitochondrial matrix
organelle inner membrane
mitochondrial inner membrane
−log10(pValue)
8
7
6
5
4
size
5.0
7.5
10.0
12.5
15.0
17.5
0.050
0.075
0.100
0.125
Enrichment Ratio
D
All geneontology_Molecular_Function
glycolipid binding
O−methyltransferase activity
snRNA binding
ribonucleoprotein complex binding
−log10(pValue)
4.50
4.25
4.00
3.75
3.50
3.25
size
3
4
5
6
7
0.03
0.04
0.05
Enrichment Ratio

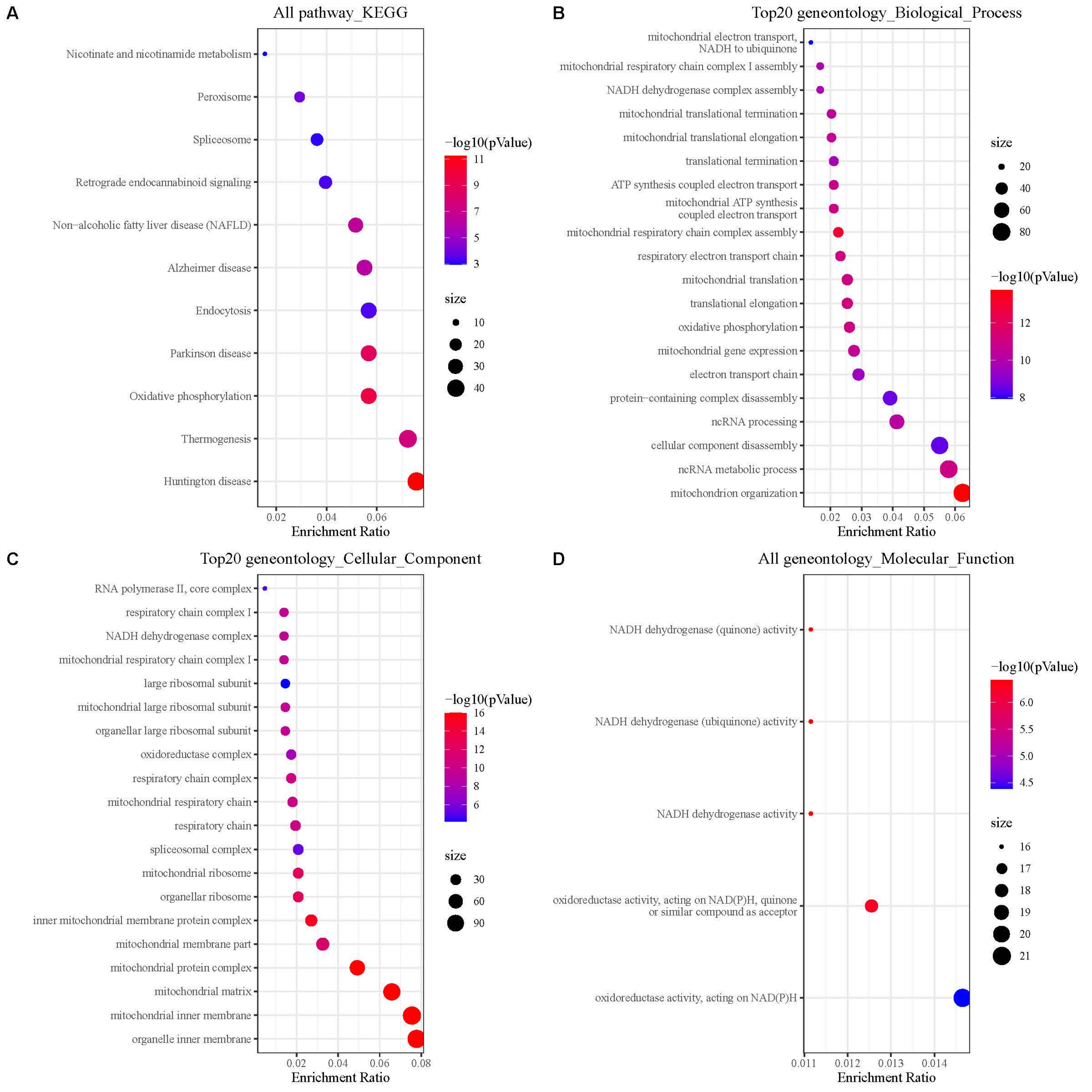
A
All pathway_KEGG
Nicotinate and nicotinamide metabolism
Peroxisome
Spliceosome
Retrograde endocannabinoid signaling
Non−alcoholic fatty liver disease (NAFLD)
Alzheimer disease
Endocytosis
Parkinson disease
Oxidative phosphorylation
Thermogenesis
Huntington disease
0.02
0.04
0.06
Enrichment Ratio
−log10(pValue)
11
9
7
5
3
size
10
20
30
40
B
Top20 geneontology_Biological_Process
mitochondrial electron transport, NADH to ubiquinone
mitochondrial respiratory chain complex I assembly
NADH dehydrogenase complex assembly
mitochondrial translational termination
mitochondrial translational elongation
translational termination
ATP synthesis coupled electron transport
mitochondrial ATP synthesis coupled electron transport
mitochondrial respiratory chain complex assembly
respiratory electron transport chain
mitochondrial translation
translational elongation
oxidative phosphorylation
mitochondrial gene expression
electron transport chain
protein−containing complex disassembly
ncRNA processing
cellular component disassembly
ncRNA metabolic process
mitochondrion organization
0.02
0.03
0.04
0.05
0.06
Enrichment Ratio
size
20
40
60
80
−log10(pValue)
12
10
8
C
Top20 geneontology_Cellular_Component
RNA polymerase II, core complex
respiratory chain complex I
NADH dehydrogenase complex
mitochondrial respiratory chain complex I
large ribosomal subunit
mitochondrial large ribosomal subunit
organellar large ribosomal subunit
oxidoreductase complex
respiratory chain complex
mitochondrial respiratory chain
respiratory chain
spliceosomal complex
mitochondrial ribosome
organellar ribosome
inner mitochondrial membrane protein complex
mitochondrial membrane part
mitochondrial protein complex
mitochondrial matrix
mitochondrial inner membrane
organelle inner membrane
0.02
0.04
0.06
0.08
Enrichment Ratio
−log10(pValue)
16
14
12
10
8
6
size
30
60
90
D
All geneontology_Molecular_Function
NADH dehydrogenase (quinone) activity
NADH dehydrogenase (ubiquinone) activity
NADH dehydrogenase activity
oxidoreductase activity, acting on NAD(P)H, quinone or similar compound as acceptor
oxidoreductase activity, acting on NAD(P)H
0.011
0.012
0.013
0.014
Enrichment Ratio
−log10(pValue)
6.0
5.5
5.0
4.5
size
16
17
18
19
20
21

A

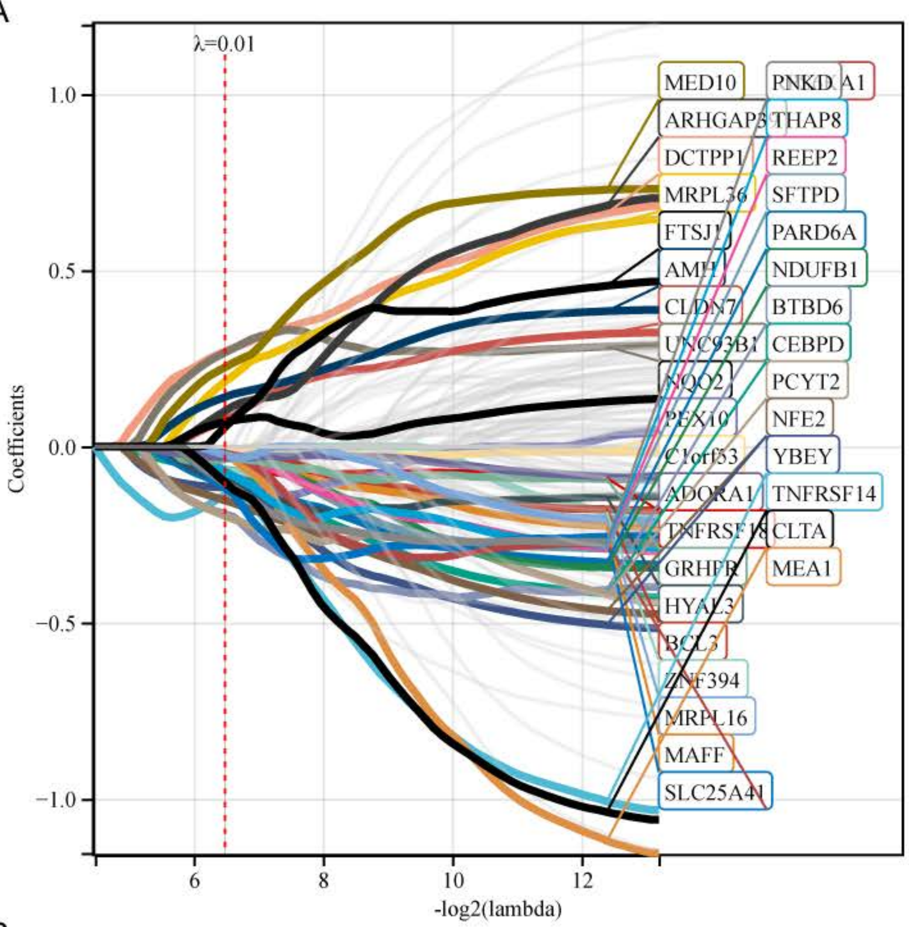

λ=0.01
Coefficients
-log2(lambda)
MED10
ARHGAP3
DCTPP1
MRPL36
FTSJ1
AMH
CLDN7
UNC93B1
NQO2
PEX10
C1orf53
ADORA1
TNFRSF18
GRHPR
HYAL3
BCL3
ZNF394
MRPL16
MAFF
SLC25A41
PNKD
THAP8
REEP2
SFTPD
PARD6A
NDUFB1
BTBD6
CEBPD
PCYT2
NFE2
YBEY
TNFRSF14
CLTA
MEA1

B

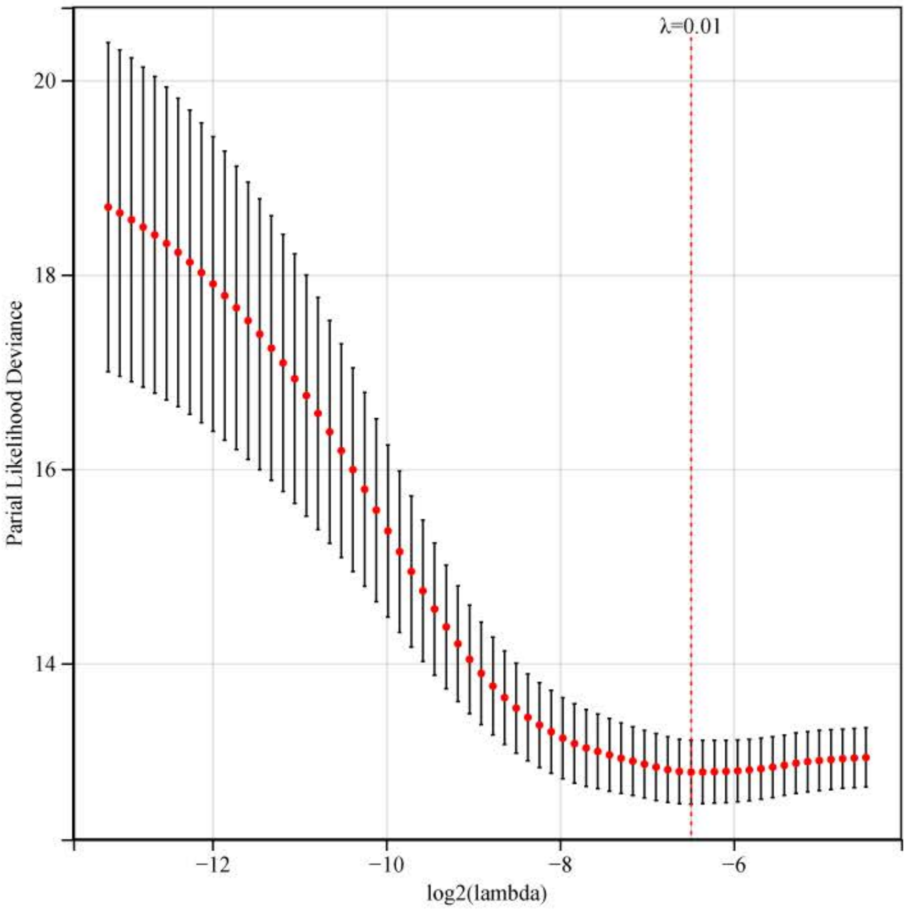

λ=0.01
Parial Likelihood Deviance
log2(lambda)

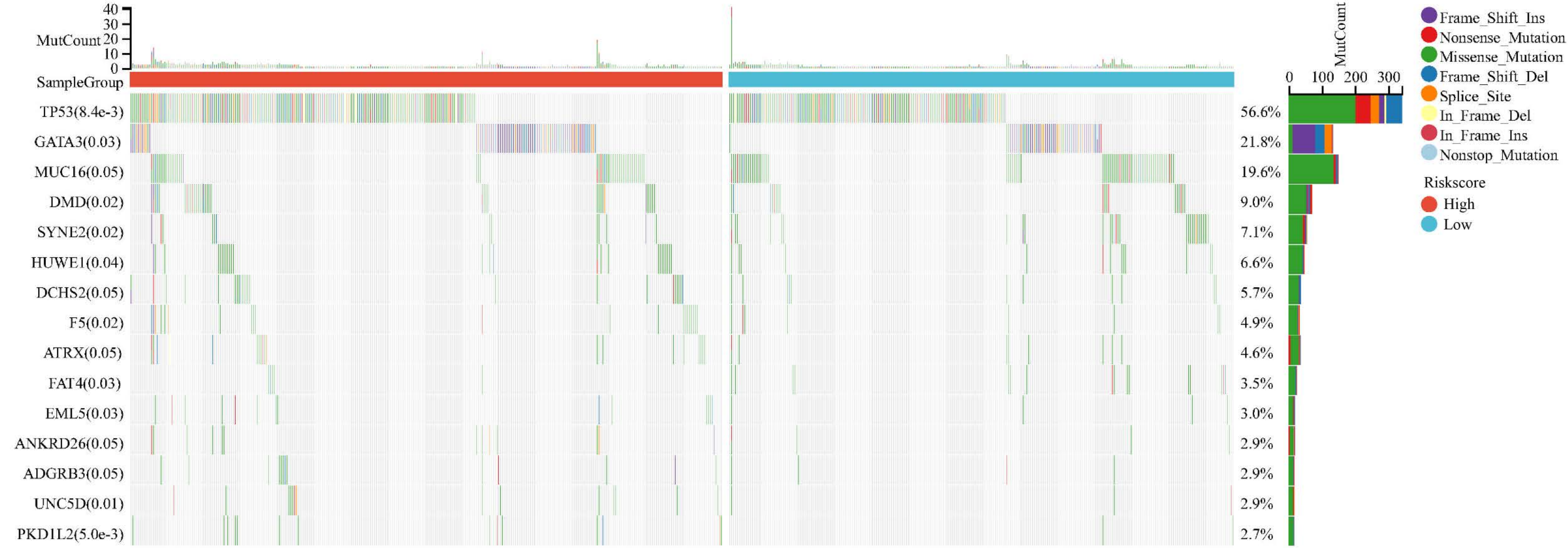
MutCount
40
30
20
10
0
SampleGroup
TP53(8.4e-3)
GATA3(0.03)
MUC16(0.05)
DMD(0.02)
SYNE2(0.02)
HUWE1(0.04)
DCHS2(0.05)
F5(0.02)
ATRX(0.05)
FAT4(0.03)
EML5(0.03)
ANKRD26(0.05)
ADGRB3(0.05)
UNC5D(0.01)
PKD1L2(5.0e-3)
56.6%
21.8%
19.6%
9.0%
7.1%
6.6%
5.7%
4.9%
4.6%
3.5%
3.0%
2.9%
2.9%
2.9%
2.7%
MutCount
0
100
200
300
Frame_Shift_Ins
Nonsense_Mutation
Missense_Mutation
Frame_Shift_Del
Splice_Site
In_Frame_Del
In_Frame_Ins
Nonstop_Mutation
Riskscore
High
Low

Supplementary Table 1. Gene set of pathway associated with immunotherapy.

Pathway	Genes
**IFN-γ_signature**	TIGIT; CD27; CD8A; PDCD1LG2; LAG3; CD274; CXCR6; CMKLR1; NKG7; CCL5; PSMB10; IDO1; CXCL9; HLA-DQA1; CD276; STAT1; HLA-DRB1; HLA-E
**APM_signal**	B2M; HLA-A; HLA-B; HLA-C; TAP1; TAP2
**Base_excision_repair**	FEN1; LIG1; NEIL3; PARP1; PARP2; PCNA; POLE; POLE2; UNG
**Cell_cycle**	BUB1; BUB1B; CCNA2; CCNB2; CCNE1; CCNE2; CDC20; CDC25A; CDC25C; CDC6; CDK1; CDK2; CDKN2A; DBF4; E2F1; E2F2; ESPL1; MAD2L1; MAD2L2; MCM2; MCM4; MCM6; MCM7; ORC1; ORC6; PCNA; PLK1; SKP2; SMC3; TFDP1; TTK; YWHAB
**DNA_replication**	DNA2; FEN1; LIG1; MCM2; MCM4; MCM6; MCM7; PCNA; POLA2; POLE; POLE2; PRIM1; PRIM2; RFC2; RFC3; RFC4; RFC5; RNASEH2A; RPA1; RPA3
**Fanconi_anemia_pathway**	BLM; BRCA1; BRCA2; BRIP1; EME1; ERCC4; FANCA; FANCB; FANCD2; FANCI; PALB2; RAD51; RAD51C; RMI1; RMI2; RPA1; RPA3; TOP3A; UBE2T
**Homologous_recombination**	BLM; BRCA2; EME1; RAD51; RAD51C; RAD54L; RPA1; RPA3; TOP3A; XRCC2; XRCC3
**MicroRNAs_in_cancer**	BRCA1; CCNE1; CCNE2; CDC25A; CDC25C; CDCA5; CDKN2A; DNMT1; E2F1; E2F2; EZH2; KIF23; STMN1; TRIM71
**Mismatch_repair**	EXO1; LIG1; PCNA; RFC2; RFC3; RFC4; RFC5; RPA1; RPA3
**Nucleotide_excision_repair**	CETN2; ERCC4; LIG1; PCNA; POLE; POLE2; RFC2; RFC3; RFC4; RFC5; RPA1; RPA3
**Oocyte_meiosis**	AURKA; BUB1; CCNB2; CCNE1; CCNE2; CDC20; CDC25C; CDK1; CDK2; ESPL1; FBXO5; MAD2L1; MAD2L2; PLK1; SGOL1; SMC3; YWHAB
**p53_signaling_pathway**	CCNB2; CCNE1; CCNE2; CDK1; CDK2; CDKN2A; GTSE1; PPM1D; RFWD2; RRM2
**Progesterone-mediated oocyte_maturation**	BUB1; CCNA2; CCNB2; CDC25A; CDC25C; CDK1; CDK2; MAD2L1; MAD2L2; PLK1
**Proteasome**	IFNG; PSMA4; PSMB2; PSMB4; PSMC4; PSMD4; PSMD7
**Pyrimidine_metabolism**	CTPS1; DTYMK; POLA2; POLE; POLE2; PRIM1; PRIM2; RRM2; TYMS
**Spliceosome**	HNRNPM; LSM3; LSM4; LSM5; MAGOHB; PRPF19; SF3B2; SF3B3; SF3B4; SNRNP40; SNRPA1; SNRPC; USP39; WBP11
**Systemic_lupus_erythematosus**	H2AFV; H2AFZ; HIST1H2AB; HIST1H2AG; HIST1H2AH; HIST1H2AI; HIST1H2AM; HIST1H2BC; HIST1H2BD; HIST1H2BF; HIST1H2BJ; HIST1H2BK; HIST1H2BL; HIST1H2BN; HIST1H2BO; HIST1H3B; HIST1H3D; HIST1H3H; HIST1H4A; HIST1H4B; HIST2H2AB; HIST2H2AC; HIST2H2BE; HIST2H2BF; HIST
**Viral_carcinogenesis**	CCNA2; CCNE1; CCNE2; CDC20; CDK1; CDK2; CDKN2A; CREB3L4; GTF2E1; HIST1H2BC; HIST1H2BD; HIST1H2BF; HIST1H2BJ; HIST1H2BK; HIST1H2BL; HIST1H2BN; HIST1H2BO; HIST1H4A; HIST1H4B; HIST2H2BE; HIST2H2BF; SKP2; YWHAB

Supplementary Table 2. Survival related genes filtered by univariate Cox regression analysis.

Tag	HR	Lower	Upper	Likelihood	logrank	Wald
TNFRSF14	0.672222	0.559277	0.807975	4.10E-05	2.69E-05	2.32E-05
CEBPD	0.778446	0.676163	0.896201	0.000456	0.000482	0.000493
UBXN11	0.724392	0.596718	0.879382	0.001046	0.001097	0.001117
YBEY	0.673315	0.530175	0.855101	0.00123	0.001212	0.00118
DCTPP1	1.506211	1.175919	1.929277	0.001003	0.001249	0.001183
NUDT14	0.714118	0.580386	0.878663	0.001675	0.001533	0.001459
BTBD6	0.62818	0.471538	0.836858	0.002244	0.001617	0.001488
ZNF394	0.509512	0.33535	0.774126	0.002006	0.001679	0.00158
HYI	0.689225	0.546161	0.869765	0.001876	0.001704	0.001716
TNFRSF18	0.835118	0.743931	0.937481	0.002064	0.002193	0.002256
UBXN1	0.710746	0.571336	0.884173	0.003096	0.002271	0.002176
MBLAC1	0.678998	0.528805	0.871848	0.00198	0.002391	0.002404
ARHGEF1	0.68954	0.541998	0.877246	0.002764	0.002628	0.002477
CCDC28B	0.719862	0.580358	0.892899	0.002361	0.002874	0.002784
NFE2	0.73804	0.60347	0.902619	0.001577	0.00295	0.003101
ZDHHC8	0.724549	0.58334	0.89994	0.004084	0.00357	0.003578
ZNF219	0.774279	0.651271	0.92052	0.003743	0.003682	0.003753
MZT2A	0.777944	0.655251	0.92361	0.004439	0.004163	0.004139
TNIP1	0.67787	0.517959	0.887152	0.005991	0.004685	0.004623
FAU	0.714015	0.565699	0.901216	0.006319	0.005102	0.004576
TMEM91	0.732493	0.586883	0.91423	0.005197	0.005815	0.005906
PCYT2	0.705437	0.548869	0.906667	0.007036	0.006811	0.006427
BCL3	0.767409	0.634078	0.928778	0.009183	0.007212	0.006553
PARD6A	0.773599	0.640936	0.933721	0.007669	0.007629	0.007486
MAFF	0.762213	0.623075	0.932423	0.007196	0.008253	0.008282
XRCC1	0.6922	0.526026	0.91087	0.010872	0.009047	0.008628
NDUFB1	0.676832	0.505035	0.90707	0.008461	0.009192	0.008979
NAB2	0.751076	0.605625	0.93146	0.009882	0.009201	0.009148
GSTT2B	0.833589	0.725997	0.957126	0.00787	0.009598	0.009839
PEX10	0.683523	0.512485	0.911643	0.010637	0.009763	0.009611
SFTPD	0.776945	0.641697	0.940699	0.00653	0.009957	0.009697
WDR83	0.706665	0.541839	0.921629	0.011258	0.010472	0.0104
C1orf53	0.793042	0.663718	0.947565	0.01022	0.010624	0.010682
BATF	0.854657	0.757476	0.964307	0.012488	0.01074	0.010769
EFHD2	0.754065	0.607448	0.936069	0.011384	0.010833	0.010502
SNAPC2	0.756093	0.609923	0.937292	0.01202	0.010994	0.010748
USF2	0.694308	0.524502	0.91909	0.012515	0.011241	0.010786
ERF	0.743205	0.59006	0.936098	0.013229	0.01185	0.011707
REEP2	0.813455	0.69175	0.956571	0.010057	0.01237	0.012528
TRMT2A	0.724447	0.563111	0.932007	0.013257	0.012453	0.01215
HINT2	0.733579	0.57448	0.936738	0.014621	0.013439	0.012995
HYAL3	0.74632	0.591714	0.941323	0.012511	0.013476	0.013491
C6orf226	0.775982	0.633982	0.949787	0.014287	0.014185	0.013911
ZBTB17	0.712018	0.54321	0.933286	0.016961	0.014274	0.013892
ZNF358	0.775703	0.633715	0.949504	0.015449	0.01436	0.013805
DDAH2	0.787093	0.649955	0.953167	0.0166	0.014378	0.014245
MEA1	0.67922	0.499541	0.923527	0.014865	0.014402	0.013608

CCDC107	0.761406	0.612135	0.947078	0.015199	0.014521	0.014352
THAP8	0.687883	0.50983	0.92812	0.014998	0.014687	0.014363
NDUFAB1	1.502098	1.083132	2.083125	0.014086	0.01513	0.014744
MIIP	0.744533	0.587107	0.944172	0.014669	0.015449	0.014935
DUS1L	0.745245	0.588143	0.944311	0.016509	0.015642	0.014919
GSTK1	0.717515	0.548028	0.939419	0.018246	0.016137	0.015756
MZT2B	0.830455	0.713758	0.966233	0.017415	0.016322	0.016192
RPS6KA1	0.74243	0.582048	0.947004	0.018566	0.016752	0.016463
MAP1LC3A	0.81095	0.682914	0.96299	0.018048	0.016926	0.016844
ERCC1	0.679499	0.495158	0.932469	0.018602	0.016945	0.016712
DAXX	0.699399	0.521985	0.937114	0.019215	0.017117	0.016618
RFNG	0.741863	0.580531	0.94803	0.019044	0.017434	0.017008
GRHPR	0.684106	0.499725	0.936515	0.019351	0.018236	0.017822
ADORA1	0.874395	0.781763	0.978003	0.016745	0.018505	0.01881
INO80B	0.717562	0.544228	0.946102	0.017998	0.018697	0.018637
SART1	0.777153	0.630198	0.958375	0.023209	0.018806	0.018397
CPSF4	0.679878	0.493287	0.937049	0.020304	0.018982	0.018414
CCDC12	0.731796	0.563867	0.949737	0.019463	0.019103	0.01889
GRINA	1.301298	1.043844	1.622251	0.019164	0.019624	0.019209
EIF3G	0.727012	0.556395	0.949948	0.021092	0.019925	0.019479
RING1	0.710835	0.533481	0.947148	0.022066	0.020043	0.019766
CADM4	0.828271	0.706283	0.971328	0.023699	0.020274	0.020458
MTA1	0.740076	0.57428	0.953738	0.022213	0.02035	0.020019
UNC93B1	1.282872	1.039222	1.583646	0.021298	0.021045	0.020451
CORT	0.562334	0.343189	0.921415	0.01872	0.022261	0.022324
AIMP2	1.441809	1.055215	1.970039	0.020586	0.022432	0.021595
PIGU	1.415374	1.051908	1.904427	0.023812	0.022547	0.021781
ACAA1	0.738121	0.568322	0.958652	0.024626	0.023031	0.022813
CLASRP	0.760096	0.600159	0.962654	0.023598	0.023041	0.022862
SLC25A41	0.575668	0.357214	0.927719	0.017273	0.02318	0.023322
ODF3B	0.829113	0.705168	0.974845	0.022402	0.023444	0.023308
EML3	0.745776	0.577497	0.963091	0.027134	0.025027	0.024563
SIRT7	0.726583	0.548639	0.962241	0.026515	0.026202	0.025846
NDUFB7	0.784412	0.634315	0.970027	0.029437	0.027025	0.025039
SNRNP70	0.759707	0.595405	0.969348	0.026631	0.027283	0.027081
FADS3	0.770675	0.611517	0.971256	0.026801	0.027371	0.027309
AGTRAP	0.767442	0.606604	0.970925	0.029354	0.027728	0.027394
KCTD17	0.780673	0.626225	0.973213	0.028843	0.027848	0.02771
IRF7	0.84495	0.72718	0.981793	0.029826	0.027999	0.027815
MRPL36	1.349434	1.033761	1.761503	0.030655	0.0284	0.027512
ZRSR2	0.701773	0.511705	0.962439	0.031874	0.028715	0.027983
PNKD	0.771933	0.61231	0.973168	0.029047	0.028766	0.028518
ZBTB48	0.72248	0.539623	0.9673	0.030387	0.029302	0.029016
CHMP6	0.766688	0.603452	0.97408	0.034079	0.03015	0.029634
SERTAD1	0.780253	0.623903	0.975785	0.031325	0.030189	0.029644
AKAP8L	0.72908	0.548049	0.969908	0.030992	0.030353	0.030023
RFXANK	0.752898	0.582863	0.972535	0.031447	0.030433	0.029765
PICK1	0.759454	0.591992	0.974286	0.031782	0.030538	0.030393
TECPR1	0.725941	0.543174	0.970205	0.031514	0.030837	0.030436

CLDN7	1.24838	1.021356	1.525865	0.024698	0.031628	0.030287
SNRNP35	0.718713	0.531827	0.971272	0.033219	0.031691	0.031581
RBFA	0.728651	0.545803	0.972755	0.032339	0.031836	0.031766
SMARCD3	0.826578	0.694361	0.983972	0.031483	0.032101	0.032221
USE1	0.735741	0.556017	0.973558	0.033038	0.032253	0.031754
CCDC106	0.825888	0.693287	0.98385	0.034201	0.032258	0.032169
DMAP1	0.716528	0.527609	0.973092	0.033825	0.032875	0.032791
POLR2I	0.731616	0.549265	0.974506	0.032633	0.033425	0.032637
MTFP1	0.776317	0.614614	0.980562	0.034115	0.033671	0.033613
GPX1	0.774997	0.612563	0.980503	0.038799	0.034786	0.033667
MRPL16	0.700146	0.503553	0.973491	0.036884	0.035226	0.034029
AMH	1.149816	1.009178	1.310054	0.041046	0.035642	0.035975
SDHA	1.364184	1.022137	1.820694	0.035787	0.03646	0.034976
MED10	1.399937	1.020894	1.919714	0.036651	0.03747	0.036769
TMEM160	0.81197	0.667351	0.98793	0.036849	0.037906	0.037406
RBM42	0.769873	0.601228	0.985823	0.040879	0.038934	0.038159
PNKP	0.76304	0.590483	0.986023	0.040658	0.039019	0.03868
ARHGAP39	1.245208	1.010813	1.533957	0.039945	0.039411	0.0393
ANAPC11	0.785568	0.624857	0.987613	0.041754	0.039711	0.038763
NAT14	0.825918	0.6882	0.991195	0.040412	0.039963	0.03988
TRIP6	0.855653	0.737157	0.993198	0.045419	0.0402	0.040393
COPS6	0.718184	0.524676	0.983061	0.045551	0.04055	0.03877
SARS2	0.719046	0.523689	0.98728	0.040891	0.041783	0.041439
ZGPAT	0.803744	0.651088	0.992192	0.041302	0.042322	0.042062
FKBP2	0.844914	0.718083	0.994145	0.047356	0.042496	0.042283
ASPSCR1	0.763798	0.588637	0.991082	0.042726	0.042748	0.042625
PPFIA3	1.195257	1.004555	1.422161	0.047307	0.044226	0.044304
PAOX	0.773854	0.602842	0.993377	0.046761	0.044525	0.044208
CROCC	0.813886	0.665633	0.995158	0.045652	0.044681	0.044721
MPST	0.796733	0.638188	0.994666	0.046178	0.045167	0.044728
TRMT1	0.769954	0.596087	0.994536	0.045428	0.045538	0.045293
EMID1	0.868412	0.755649	0.998002	0.043456	0.046607	0.046795
FTSJ1	1.479993	1.007408	2.174272	0.044849	0.046982	0.045763
CNPY3	0.743532	0.555507	0.995198	0.049838	0.04763	0.046335
IFI27L1	0.784307	0.616818	0.997275	0.047468	0.047726	0.047454
NQO2	1.322097	1.002061	1.744344	0.049821	0.048581	0.048322
PKN3	0.811458	0.659237	0.998829	0.047237	0.048693	0.048722
NDUFAF3	0.772568	0.597854	0.998339	0.051353	0.049151	0.048533
PRKCZ	0.769242	0.592092	0.999396	0.052593	0.049661	0.049473
CLTA	0.716551	0.514934	0.997108	0.050968	0.049766	0.048026
ABHD8	0.794914	0.631948	0.999906	0.052056	0.049943	0.049906
CCDC9	0.803602	0.645804	0.999956	0.055765	0.049949	0.049954
